# Evaluation of the comorbidity burden in patients with ankylosing spondylitis treated with tumour necrosis factor inhibitors using a large administrative claims data set

**DOI:** 10.1111/jphs.12212

**Published:** 2018-01-16

**Authors:** Jessica A. Walsh, Xue Song, Gilwan Kim, Yujin Park

**Affiliations:** ^1^ University of Utah and Salt Lake City Veteran Affairs Medical Center Division of Rheumatology Salt Lake City UT USA; ^2^ Truven Health Analytics, an IBM Company Cambridge MA USA; ^3^ Novartis Pharmaceuticals Corporation East Hanover NJ USA

**Keywords:** outcomes research, pharmaceutical HSR

## Abstract

**Objectives:**

Comorbidity incidence rates among US patients with ankylosing spondylitis (AS) treated with tumour necrosis factor inhibitors (TNFis) are inadequately understood. This study compared the relative occurrence of comorbidities between patients with AS treated with TNFis and those not treated with TNFis.

**Methods:**

Adults aged ≥18 years enrolled in the MarketScan Commercial and Medicare Supplemental databases with a diagnosis of AS between 1 January 2008 and 30 June 2015 were eligible. Patients were divided into two groups, those treated with TNFis (TNFi users) and those not treated with TNFis (TNFi nonusers) during the 12 months after the index date, defined as the date of first TNFi treatment or a randomly assigned date for TNFi nonusers. Patients had to have continuous enrolment for 24 months with no AS diagnosis or TNFi therapy pre‐index and a follow‐up period of ≥12 months postindex. The incidence of new comorbidities was evaluated in patients and adjusted for baseline characteristics.

**Key findings:**

A total of 3077 TNFi users and 3830 TNFi nonusers were included. A higher proportion of TNFi users had a new diagnosis of inflammatory bowel disease (hazard ratio [HR], 2.00), including Crohn's disease (HR, 2.45) and ulcerative colitis (HR, 1.65), as well as uveitis (HR, 1.68) and sleep apnoea (HR, 1.21) after initiation of TNFi therapy than TNFi nonusers.

**Conclusions:**

Patients with AS treated with TNFis had higher incidence rates of IBD, uveitis and sleep apnoea after initiation of TNFi therapy than patients not treated with TNFi therapy.

## Introduction

Ankylosing spondylitis (AS), a systemic inflammatory disorder known to affect approximately 0.1–1% of the general population, affects the axial skeleton,^1^, ^2^, ^3^, ^4^ peripheral joints, entheses, eyes, skin, intestine and cardiovascular system^5^, ^6^, ^7^; AS occurs more frequently in men, with a higher prevalence in white patients than in non‐white patients.^8^, ^9^ The typical age of onset ranges from the late teenage years through 40 years of age. Although AS onset after 50 years of age is unusual, delays in diagnoses are known to occur and have been estimated to be 8–11 years in some individuals; these delays may contribute to diagnoses occurring at older ages.^10^, ^11^

In recent years, biologic treatment, specifically with tumour necrosis factor inhibitors (TNFis), has been found to be an effective treatment for AS.^12^, ^13^, ^14^ Current treatment recommendations published by the American College of Rheumatology report strong evidence supporting treatment with TNFis along with nonsteroidal anti‐inflammatory drugs (NSAIDs) when treatment with NSAIDs alone is insufficient.^15^ In 2003, etanercept, a TNF‐α blocker, became the first biologic disease‐modifying antirheumatic drug to be approved for treatment of AS by the US Food and Drug Administration (FDA).^12^ In the last decade, four additional TNFis have been approved for treatment of AS: adalimumab, certolizumab pegol, golimumab and infliximab.^16^ In 2016, the fully human IL‐17A inhibitor secukinumab also received FDA approval for treatment of AS.^13^

Many studies, conducted mostly outside the USA, have found that patients with AS have a higher risk for comorbidities, including hypertension, hyperlipidemia, diabetes mellitus, peptic ulcers, headaches, depression, uveitis, cancer, inflammatory bowel disease (IBD: Crohn's disease, ulcerative colitis), osteoporosis, psoriasis, and other cardiovascular, pulmonary, renal and neurological complications.^17^, ^18^, ^19^, ^20^, ^21^, ^22^, ^23^, ^24^, ^25^, ^26^, ^27^ Very few studies have examined comorbidities in patients with AS in the USA using real‐world data, and still fewer have examined comorbidities in patients receiving specific therapies.

To better understand the associations between newly diagnosed comorbidities and TNFi use, this study compared incidence rates of new comorbidities among patients with AS treated with TNFis versus those not treated with TNFis from a large, US healthcare claims database.

## Methods

### Data sources

This retrospective, observational analysis used healthcare claims from two large administrative claims databases, the Truven Health MarketScan Commercial Claims and Encounters (Commercial) database and the MarketScan Medicare Supplemental (Medicare) database, from 1 January 2008 through 30 June 2015. Both databases include complete longitudinal records of inpatient services, outpatient services, long‐term care and prescription drug claims for commercially insured and Medicare‐eligible patients covered under a variety of health plans. All pharmacy fills with health plan payment or patient copayment are included in the claims database; unfilled prescriptions are not included. Demographic data, diagnostic codes, prescribing physician specialty and type of insurance plan are recorded at the time of prescription fill. In 2014, the Commercial and Medicare Supplemental databases included data on approximately 38 million and 3.5 million covered patients respectively. All study data were accessed with protocols compliant with US patient confidentiality requirements, including the Health Insurance Portability and Accountability Act of 1996 regulations. As this study used only statistically de‐identified patient records, it was exempted from institutional review board approval.

### Patient selection

Adult (aged ≥ 18 years) patients with AS were required to have an AS diagnosis (International Classification of Diseases, Ninth Revision, Clinical Modification (ICD‐9‐CM) diagnosis code 720.0) in any position on ≥1 inpatient or ≥2 outpatient medical claims >30 days apart but ≤365 days of each other between 1 January 2008 and 30 June 2015. Patients with AS were identified as those continuously enrolled with medical and pharmacy benefits ≥24 months prior to the first observed AS diagnosis and with no evidence of AS diagnosis during the 24‐month baseline (pre‐index) period. Patients were divided into two subcohorts based on whether they had been treated with TNFis or had not received TNFi treatment (TNFi nonusers) during the 12‐month follow‐up (postindex) period. For this analysis, the index date was defined as the date of first TNFi claim for the TNFi users. The interval (number of days) between the TNFi index date and the first AS diagnosis date was calculated for all TNFi users (‘interval pool’). For the TNFi nonusers, an interval was randomly selected from the interval pool and added to their first AS diagnosis date. The resulting date was assigned as the index date for the TNFi nonuser. All patients were followed up for ≥12 months after their index date until the earliest of inpatient death, end of continuous enrolment or end of the study period.

### Study variables

Patient demographic data recorded on the index date included age, sex, geographic region of residence, type of health plan and duration of follow‐up. The comorbidities of interest were detected by the presence of a diagnosis code on a medical claim, but excluded diagnostic or rule out procedures (e.g. laboratory, pathology or radiology services) to avoid incorrectly identifying patients as having a comorbidity based on the history of testing rather than the test results. The following comorbidities were measured during the baseline and follow‐up periods: cardiovascular conditions (e.g. angina, atherosclerosis, cerebrovascular disease/stroke, coronary artery disease, hypertension, myocardial infarction, peripheral vascular disease and venous thromboembolism), IBD (Crohn's disease or ulcerative colitis), gastrointestinal ulcers (oesophageal, gastric, duodenal, peptic or gastrojejunal), malignant neoplasms, diabetes, dyslipidaemia, multiple sclerosis, Parkinson's disease, depression, asthma, sleep apnoea, osteoporosis and uveitis. ICD‐9‐CM codes used for these conditions are available upon request. The mean Deyo‐Charlson Comorbidity Index score was reported based on comorbidities measured in the baseline period.

Individual flags for comorbidities of interest were created during the 24‐month baseline period and the variable‐length follow‐up period. A comorbidity was considered newly diagnosed in the follow‐up period if it was not reported during the baseline period.

Frequencies of comorbidities were reported for TNFi users and TNFi nonusers. In addition, incidence rates per 100 patient‐years for new comorbidities were calculated during the variable‐length follow‐up period. The incidence rate was calculated as ([number of patients with specific comorbidity of interest in follow‐up period/total observation days]/365) × 100. The total observation days were accumulated from the index date to the date of the first observed comorbidity for patients with the new comorbidity or the entire follow‐up period for patients without the specific comorbidity. Patients with the specific comorbidity in the baseline period were excluded from the incidence rate calculation.

### Analyses

All study variables, including demographic characteristics and baseline comorbidities, were described separately for TNFi users and TNFi nonusers. Categorical variables were summarized by counts and percentages, and continuous variables were summarized with means and standard deviations. χ^2^ tests were used to compare categorical measures, and Student's *t*‐tests were used for continuous measures. The threshold for statistical significance was set *a priori* to the *P* value of 0.05.

Cox proportional hazards models were estimated to examine the difference in the risk of developing a comorbidity between TNFi users and TNFi nonusers. Hazard ratios (HRs) were adjusted for patients' demographic characteristics and baseline comorbidities. All analyses were conducted using SAS version 9.4 (SAS Institute Inc, Cary, NC, USA).

## Results

### Study population

**Figure 1:**
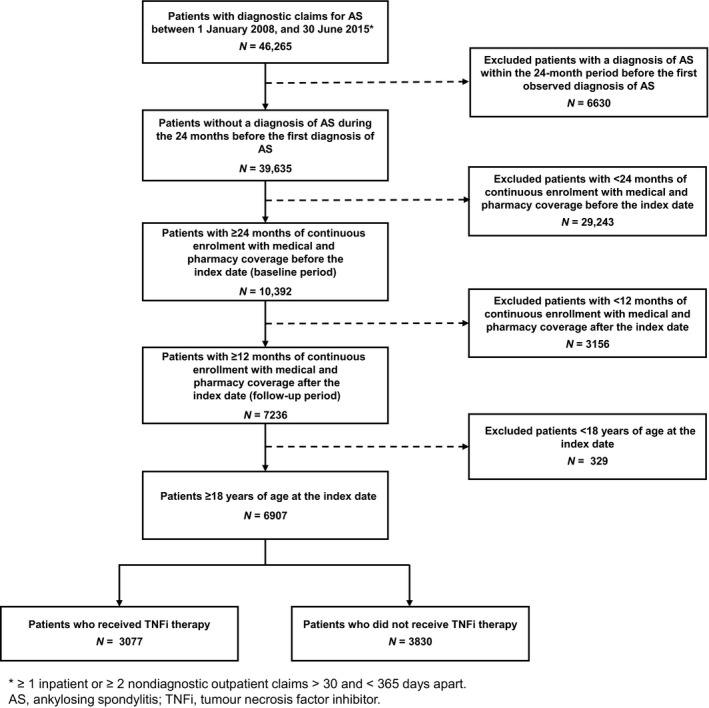
Patient selection.

Of the 153 million individuals included in the MarketScan databases from 1 January 2008 to 30 June 2015, a total of 46 265 patients had AS; among them, 6907 met all the study criteria, with 3077 treated with TNFi therapy (TNFi users) and 3830 not treated with TNFi therapy (TNFi nonusers) (Figure 1).

### Patient demographic characteristics and baseline comorbidities

On average, TNFi users were ≈8 years younger than TNFi nonusers (mean [SD], 46.6 (13.3) versus 55.0 (14.9) years; *P *< 0.001). More than half the patients in both cohorts were men and were enrolled in a preferred provider organization health plan. Both cohorts had similar lengths of follow‐up periods (mean, 1000–1032 days; Table 1).

**Table 1:** Patient demographic characteristics at the index date in TNFi users and TNFi nonusers

Demographic characteristics	TNFi users	TNFi nonusers	*P* value
	*N *=* *3077	*N *=* *3830	
Age, mean (SD), years	46.6 (13.3)	55.0 (14.9)	<0.001
Age group, %			<0.001
18–34 years	18.4	9.1	
35–44 years	23.9	14.2	
45–54 years	28.2	23.7	
55–64 years	22.8	30.0	
65+ years	6.6	23.1	
Male, %	54.5	55.2	0.535
Geographic region, %			<0.001
Northeast	15.8	16.9	
North central	20.3	23.3	
South	38.1	31.0	
West	25.4	28.3	
Unknown	0.4	0.4	
Health plan type, %			<0.001
PPO	58.0	55.4	
HMO	15.1	15.1	
Comprehensive	4.8	12.0	
POS	9.1	6.8	
Other	13.0	10.7	
Length of follow‐up, mean (SD), days	1032 (573)	1000 (568)	0.020

HMO, health maintenance organization; POS, point‐of‐service plan; PPO, preferred provider organization; TNFi, tumour necrosis factor inhibitor.

TNFi users had significantly lower overall comorbidity (as measured by the Deyo‐Charlson Comorbidity Index) than TNFi nonusers (mean [SD], 0.71 [1.14] versus 0.99 [1.60]; *P *<* *0.001), and significantly lower proportions of TNFi users versus TNFi nonusers had cardiovascular disorders, dyslipidaemia, diabetes, malignancies, osteoporosis and Parkinson's disease during the baseline period. TNFi users also had significantly higher baseline rates of depression (15.5% versus 13.0%; *P *=* *0.002), IBD (10.0% versus 4.1%; *P *<* *0.001) and uveitis (12.9% versus 10.4%; *P *=* *0.002) than TNFi nonusers (Table 2).

**Table 2:** Baseline comorbidities in TNFi users and TNFi nonusers

Baseline comorbidities	TNFi users *N *=* *3077	TNFi nonusers *N *=* *3830	*P* value
Deyo‐Charlson Comorbidity Index, mean (SD)	0.71 (1.14)	0.99 (1.60)	<0.001
Cardiovascular, %	36.2	48.9	<0.001
Angina	1.7	2.4	0.069
Atherosclerosis	6.4	13.1	<0.001
Cerebrovascular disease/stroke	2.3	4.0	<0.001
Coronary artery disease	4.4	9.4	<0.001
Hypertension	32.5	44.5	<0.001
Myocardial infarction	1.1	2.9	<0.001
Peripheral vascular disease	2.3	4.4	<0.001
Venous thromboembolism	1.7	3.1	<0.001
Dyslipidaemia, %	24.2	34.1	<0.001
Depression, %	15.5	13.0	0.002
Uveitis, %	12.9	10.4	0.002
Sleep apnoea, %	10.9	9.7	0.119
Inflammatory bowel disease, %	10.0	4.1	<0.001
Diabetes, %	9.1	13.2	<0.001
Malignancies, %	5.6	12.1	<0.001
Osteoporosis, %	3.9	5.9	<0.001
Asthma, %	3.4	3.7	0.476
Gastrointestinal ulcers, %	1.5	1.6	0.744
Multiple sclerosis, %	0.4	0.5	0.769
Parkinson's disease, %	0.1	0.5	0.006

AS, ankylosing spondylitis; TNFi, tumour necrosis factor inhibitor.

### Study outcomes

**Figure 2:**
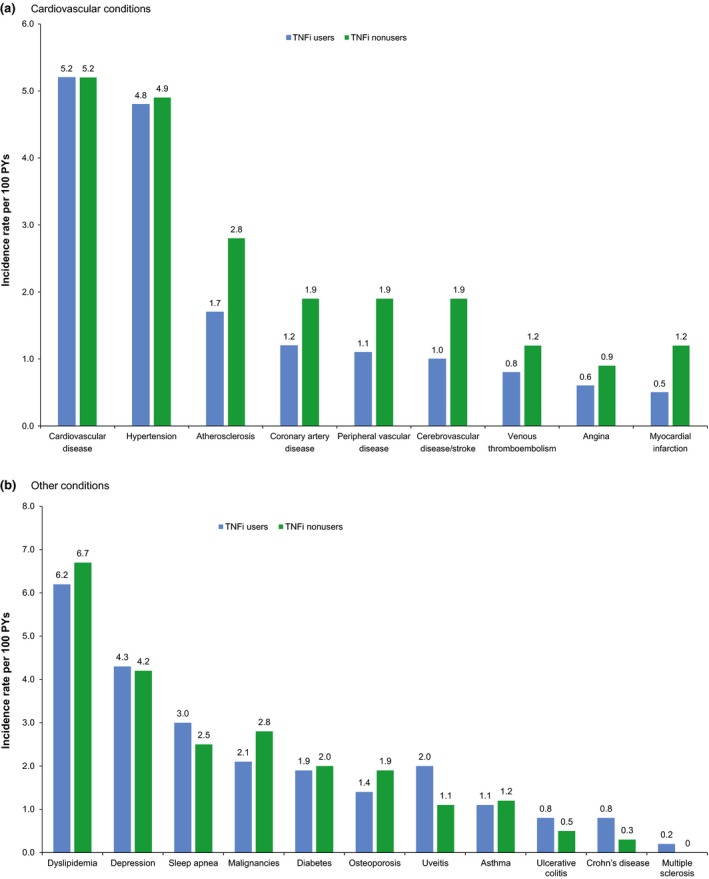
Incidence rates per 100 PYs of new comorbidities in TNFi users versus nonusers with AS (a) Comorbidities (b) Cardiovascular comorbidities. AS, ankylosing spondylitis; PY, patient‐year; TNFi, tumour necrosis factor inhibitor.

Unadjusted analysis of newly diagnosed AS comorbidities (diagnosed in the follow‐up period and not during the 24‐month baseline period) found that TNFi users had higher incidence rates of depression, sleep apnoea, uveitis, ulcerative colitis and Crohn's disease than did TNFi nonusers (Figure 2a) and lower incidence rates of dyslipidaemia, malignancies, osteoporosis, atherosclerosis, coronary artery disease, peripheral vascular disease, cerebrovascular disease/stroke, venous thromboembolism, angina and myocardial infarction than TNFi nonusers (Figure 2a,b). TNFi users and TNFi nonusers had similar incidence rates of depression, diabetes, asthma, multiple sclerosis, cardiovascular disease and hypertension.

### Multivariate analysis

After adjusting for patient demographic characteristics and baseline comorbidities, the multivariate analyses showed that TNFi treatment was associated with a significantly higher risk of IBD (HR, 2.00; 95% CI, 1.43–2.81), including Crohn's disease (HR, 2.45; 95% CI, 1.58–3.80) and ulcerative colitis (HR, 1.65; 95% CI, 1.12–2.43), uveitis (HR, 1.68; 95% CI, 1.31–2.16), and sleep apnoea (HR, 1.21, 95% CI, 1.00–1.46) (Table 3).

**Table 3:** Adjusted hazard ratios for newly diagnosed comorbidities in TNFi users versus TNFi nonusers

Newly diagnosed comorbidities	TNFi users versus TNFi nonusers			
	Hazard ratio^a^	Lower 95% CI	Upper 95% CI	*P* value
Inflammatory bowel disease	2.00	1.43	2.81	<0.001
Crohn's disease	2.45	1.58	3.80	<0.001
Ulcerative colitis	1.65	1.12	2.43	0.012
Uveitis	1.68	1.31	2.16	<0.001
Sleep apnoea	1.21	1.00	1.46	0.046
Diabetes	1.19	0.96	1.49	0.114
Asthma	1.07	0.80	1.43	0.627
Dyslipidaemia	1.06	0.93	1.20	0.397
Osteoporosis	1.06	0.83	1.35	0.642
Hypertension	1.04	0.90	1.21	0.551
Depression	1.01	0.87	1.18	0.865
Cardiovascular disease	0.98	0.85	1.13	0.799
Malignancy	0.97	0.79	1.18	0.746

a Risk of newly diagnosed comorbidity for TNFi users relative to TNFi nonusers adjusted for patient demographic characteristics (age, gender, geographic region, health plan type and urban versus rural location) and baseline comorbidities.

TNFi, tumour necrosis factor inhibitor.

## Discussion

The primary finding from this study was the association between TNFi treatment and a higher risk for developing IBD (including Crohn's disease and ulcerative colitis), uveitis and sleep apnoea after the initiation of TNFi therapy. It is not possible to establish the cause‐and‐effect relationship between a patient's clinical condition and particular treatments; thus, observed relationships should be considered associative rather than causal. Therefore, our results do not necessarily imply that receiving TNFi therapy had a causal relationship with comorbidities. For example, it is unknown if comorbidity differences between TNFi users and nonusers are attributable to the effects of the drugs or to patient characteristics that influence decisions to use the drugs. Healthcare providers might be influenced in favour of TNFis in patients with symptoms of undiagnosed uveitis or IBD at the time when treatment is prescribed; patients with more severe AS could be selected for TNFi therapy, and there might be association between AS severity and uveitis or IBD.^28^ In addition, sleep apnoea is strongly associated with obesity, which may potentially influence symptom severity and treatment decisions.^29^, ^30^ Thus, it is possible that obesity is influencing both the selection of TNFi therapy and the risk of sleep apnoea.

The baseline data demonstrate that compared with TNFi nonusers, TNFi users had a lower comorbidity burden, with lower Deyo‐Charlson Comorbidity Index scores and significantly lower proportions of patients for most measured comorbidities. TNFi users were 8 years younger than TNFi nonusers (46.6 versus 55.0 years) at the index date. This may be due to increased comorbidities in older patients, which may prevent administration of TNFis. In addition, younger patients may have attitudes or perceptions that make them more likely to try TNFi therapies. The lower baseline comorbidity profile and younger age of TNFi users raise the possibility of selection bias for TNFi treatment in younger, healthier patients.

The mean age of patients included in the study was older than reported in previous studies of patients with AS,^31^ especially for TNFi nonusers. This may have been due to a limitation of the inclusion criteria which required continuous enrolment for 24 months before the index date. Younger patients may be more likely to switch jobs^32^ and thus switch insurance carriers. In addition, younger people may access healthcare providers less frequently and have fewer opportunities for an AS or comorbidity diagnosis. This study may have also inadvertently captured patients with established AS who did not have a claim for AS in the preceding 24 months.

The high proportion of women included in this study was also unexpected. Patients may have claims for AS, but had nonradiographic or peripheral spondyloarthritis (SpA) without AS (there are no specific diagnostic codes for these subtypes). As women more frequently have nonradiographic SpA and peripheral SpA than men,^33^ women may have been misclassified as having AS more frequently than men.

The follow‐up incidence rate data show the frequencies of newly diagnosed comorbidities after the initial AS diagnosis. Knowledge of the frequency and risk of these comorbidities may assist with comorbidity screening strategies in patients with AS. However, the unadjusted baseline comorbidity analyses (Table 2) do not account for the differences in age or other baseline characteristics between the TNFi users and nonusers. Because these baseline characteristics also influence comorbidity risk, the differences in the unadjusted baseline comorbidity rates between the TNFi users and nonusers may be attributable to factors other than TNFi use.

### Limitations

This study has several limitations inherent to using administrative claims data. Patients with AS were identified, and variables were captured through administrative claims data; therefore, AS severity and manifestations that were not recorded as a diagnosis on a medical claim were not captured. Patients with AS may not get an AS diagnosis code at every office visit, so no AS diagnosis for 24 months before the index date does not necessarily guarantee that the patients are newly diagnosed with AS. Claims data indicate receipt of a medication, but they do not include information on whether the patient used the medication as prescribed. Furthermore, claims data do not capture a patient's clinical response to TNFi treatment. Due to the large cohort sizes, small differences between comparator groups may be found to be statistically significant, although the clinical significance may be questionable. Finally, findings from this study may be prone to bias from nonrandom selection into the treatment group and were limited to patients with AS covered by Commercial and Medicare health plans; therefore, the results may not be generalizable to those covered under other types of insurance or who lack coverage.

Despite these limitations, this study was unique as it evaluated a large sample of patients from two large insurance claims databases, and it provides a contemporary update on the comorbidity burden among patients with AS treated with and without TNFi therapy. In addition, as the Commercial and Medicare databases include adult patients with AS treated by clinicians across all US geographic regions and covered under various health plans, the findings of this study reflect the status of comorbidities among patients with AS in a real‐world setting.

## Conclusions

Within a large, real‐world data set, patients with AS who were treated with TNFis had higher incidence rates of newly diagnosed IBD (including Crohn's disease and ulcerative colitis), sleep apnoea and uveitis than did patients not treated with TNFis. These associations could potentially affect strategies for screening and managing treatment of comorbidities in US patients with AS. These findings may also guide future research characterizing the relationships between comorbidity risk, AS severity and TNFi therapies.

## **Declarations**

### Conflict of interest

Xue Song and Gilwan Kim are employees of Truven Health Analytics, an IBM Company, which was paid by Novartis Pharmaceuticals Corporation in connection with the development of this manuscript. Yujin Park is an employee of Novartis. Jessica Walsh is a paid consultant to Novartis.

### Funding

This study was sponsored by Novartis Pharmaceuticals Corporation, East Hanover, NJ, USA.

### Authors' contributions

All Authors state that they had complete access to the study data that support the publication.
